# From Our 'Foreign' Correspondent

**Published:** 1983-04

**Authors:** 


### From Our "Foreign" Correspondents

### A Visit to Egypt

Sohag is a city on the Nile in Upper Egypt about halfway between Luxor and Assiut. It has a new 400 bed hospital finished about a year ago. It is affiliated to the University of Assiut for post-graduate teaching and the Professor of Surgery there is Michel Guirgis. In 1960 Michel Guirgis worked for a year at Southmead Hospital as SHO in Urology. In 1961 he obtained the Fellowships of the Royal Colleges of both England and Edinburgh. In all he spent about 4 years in England and worked also at Newcastle, Durham, Basildon and Walsall. In 1978 he paid another visit to England and Southmead Hospital where I renewed his acquaintance. He was keen to see general surgery and spent several days with us. The following year he arranged for one of his assistant surgeons Dr. Nabil Attillah to spend a couple of weeks with me at Southmead Hospital and join in the work of the firm. We were quickly impressed by his ability as well as by his command of the English language. He brought with him a colleague, Dr Attia Salama, an anaesthetist, who was taken under the wing of the Department of Anaesthetics by Dr. John Powell.

In April 1982, after 10 memorable days on a Nile cruise seeing the antiquities and monumental temples of Egypt, I took the opportunity of making a visit to Sohag to renew old friendships and to see something of life and work in an Egyptian provincial hospital based on a rural community of about 150,000. At the end of the last War I had worked for a spell of 2 years as surgeon with Arabs in Amman and greatly enjoyed the experience. I looked forward to repeating it and practising again some clinical Arabic—alas, very little of this found its way out of the memory bank, in part perhaps because Egyptian Arabic differs considerably from Jordanian.

About 6 months before my visit Dr. Nabil Attillah had been in England and came to Bristol to see me and discuss arrangements. He was there with Professor Guirgis to meet me at Sohag railway station as I got out of the train from Cairo. He had spent most of the intervening time in prison. A few weeks after returning from England he had been swept up in the mass arrests ordered by President Sadat a month before his assassination. Over a thousand people were arrested including the Coptic Pope and 8 Coptic Bishops. He was given no reason for his arrest, had been kept in internment for 5 months and only released the previous week. He had been well treated. Dr Attillah is a Copt. Sadat's reason for arresting so many Copts is not clear because they are a non-political religious group who would have nothing to gain by Sadat's removal and generally supported the foreign policies which had gained him enemies amongst the fanatical Muslims who were the main bulk of the internees. It may be that these 'harmless' Copts were included to make Sadat's arrests seem fairer and not directed exclusively against one section of the community. The Copts are descended from the Egyptians of Pharaonic times, conversion to Christianity began in the second century A.D., they suffered persecution until Christianity became the official religion of the Roman Empire in the reign of Constantine in the fourth century. After the Arab conquest in the seventh century they resisted conversion to Islam. Like some other religious minorities they are prominent in commerce and the professions and this may excite animosity. Egypt throughout its history has a fair record of religious tolerance, nevertheless the Copts are aware of the need to be very correct in their affairs. On the first morning after my arrival Professor Guirgis took me to the office of the Provincial Governor and presented me to him, he was a former Chief of Police in Cairo and a formidable man, but he had a twinkle in his eye and was courteous and friendly. Afterwards he took me to the Police Headquarters to show my passport and be registered. Later I wanted to take a photograph of him standing outside the hospital but he asked me not to as he had not had permission. Egypt is certainly at the present time and understandably in a nervous state with the police very much in evidence everywhere and armed sentries on guard at all key points.

There is a free hospital service in Egypt and this includes out-patient clinics and casualty services. There is no free general practitioner service. Salaries paid to medical staff are low and most of them supplement them by doing private work, the hours of work in Government Hospitals are 9 to 1 and the remainder of the day is available for this. My first visit to the hospital was a ward round. All the surgical beds, 48 male and 24 female were under the care of the professor. The wards each contained 6 beds, though in some cases in the female wards there were 2 patients to a bed. During the day there was one nurse in charge of each ward and at night a nurse would look after 2 or 3 wards. Dr Nemir, another consultant surgeon on the surgical unit is a Fellow of the English College of Surgeons and had worked for some years at the Central Middlesex Hospital. I also met the consultants in Urology and Orthopaedics. Also on the ward round was Dr Louis Abadir the Radiologist, a keen clinician as well as an expert radiologist and Dr Elias, a consultant physician with a gastro-enterological interest of whom more later. The cases in the surgical wards were very varied—

the familiar hernias, appendicitis, gallstones, peptic ulcers, abdominal malignancies, gangrenous legs—arteriosclerotic, diabetic and embolic. Less familiar was the abdominal tuberculosis, bladder cancer due to bilharzia, acute perforations of the ileum due to typhoid and there was one survivor after surgery for an acute perforation of an amaebic ulcer of the colon, a condition generally regarded as uniformly fatal. There was much evidence of trauma, blunt injuries, stab wounds and gunshot wounds. Inevitably also the sort of cases one seldom sees except in the photographs in the older textbooks—I am thinking particularly of a woman, the side of whose face was eaten away by a malignant tumour and a gaping hole into her mouth constantly discharged saliva.

The long white gown worn by most of the rural population in Egypt does equally well for bed wear and even for a theatre gown. They also seem reluctant to remove their turban in bed and these may even find their way into the operating theatre. It is not easy apparently to get recruits of quality into the Nursing profession though I was told that the Moslem religion does not offer any obstacle. I was also told that in private hospitals in Cairo a nurse can earn as much as four times the salary of a doctor and that young doctors work there as nurses. There is a Nursing School in Sohag where textbooks and instruction are in Arabic in contradistinction to the Medical Schools where the textbooks and the teaching are in English. The Theatre Superintendant is a Swiss Nun, and there are four other Swiss Nursing Sisters, they live alongside the other nurses and go home once in four years.

I escaped the fate that often befalls visiting surgical pundits, they had not saved up their largest goitres or hernias for me to do. The operation they were most anxious to be shown was Proximal Gastric (highly selective) Vagatomy and as I had been personally instructed in this operation by its inventor, Professor David Johnston before he left Bristol, this presented no problem. They had recently acquired fibreoptic endoscopes and were using them extensively to gain experience. I joined in this activity hoping to be of some help.

It happened that my visit coincided with Holy Week and I went with my hosts to a number of Church Services. The first of these was on Palm Sunday. The Coptic ritual is of great antiquity and is very interesting. The Church was a modern one, bright clean and airy. The Altar is behind a screen as in Greek and Russian Orthodox churches. there is a central aisle to the left of which sit the men and to right the women. The service was conducted in two languages, Arabic and Coptic. The Coptic language is derived from the language of ancient Egypt but is written in the Greek script with the addition of 7 extra letters. This language was gradually replaced after the Arab conquest in the seventh century by the Arabic language and by the sixteenth century had died out except for its liturgical use. Prayers and readings were said first in Arabic from the right hand side of the choir and then, as far as I could make out, repeated in Coptic from the left hand side. This made services extremely long. The Service on Easter Eve lasted for 6 hours, though we did not stay for the whole of this time. Before midnight all the lights in the church were extinguished and lamentations on the Crucifixion were heard from behind the Altar screen. Suddenly there was jubilant singing and ringing of bells, a procession emerged bearing lamps and candles proclaiming that Christ had risen. Leading it was the priest swinging a censer and walking backwards in front of him was an acolyte bearing a large portrait of Christ. I soon realised that his handsome youth was Ayman, the elder son of Dr. Nabil Attillah, whom I had previously met as his home.

During Holy Week I was invited several times to the houses of Professor Guirgis and Dr Attillah to eat with them, their wives prepared for me beautiful food which they themselves were unable to eat because they were fasting. On Good Friday there was no routine work at the hospital and about midday I decided to wander around the town. The Mosques were full of the Muslims at their Friday prayers and there was very little traffic on the streets. It was very, very hot. Suddenly a car drew up alongside. It was Professor Guirgis who happened to be driving by. He offered me a lift back to the hostel. His voice was hoarse and he looked totally exhausted, I was worried that he might be ill until I discovered that on Good Friday fasting was total—neither food nor water were permitted.

I was taken to see three Coptic monasteries. Two of them of exceptional interest. The Monastery of St. Chenouda was founded about AD 200 and built within a Pharaonic Temple dating about BC 1500. The precinct had therefore been in continuous use as a place of worship for three and a half millennia. The other Monastery of St. Mary was in the desert and had been unoccupied for centuries. Recently a young monk had reoccupied it and begun, more or less unaided, to restore it. From the outside it appeared to be a square fortress and no doubt in past times it had needed strong defences. It had been built near the site of a Pharaonic burial ground dating from perhaps the first millenium BC. The graves had been humble ones and there were no remaining monuments above ground. Before burial the bodies had been embalmed in traditional fashion. It was the custom to bury with them jars containing the organs removed and also gold, jewelry and ornaments. At one time or another these graves had been ransacked and the valuables stolen. The bodies had just been left in the open and there they still lay, in large pieces and in little pieces. Bones and dried flesh still on them, still with their linen wrappings adhering in places—a pelvis with a thigh bone here, a rib cage there. So dry and hot is the sand that it seems no decay takes place and no birds or reptiles are interested in nibbling them. I did not see any skulls about, perhaps they had at some time been collected by the monks and reburied.

Among the consultant medical staff of the hospital were also Christians of the Protestant persuasion. The Obstetrician and Gynaecologist Dr. Gamal Elias, F.R.C.S. and his charming wife with the MRCP a consultant physician already referred to above took me to their church on Easter Day, a fine large building. The presiding pastor wore an academic style gown, there was an excellent choir singing hymns with gay tunes one of which bore a stirking resemblance to Wonderful, Wonderful Copenhagen. At one point a member of the congregation stood up and started to speak and I recognised Dr Lois Abadir, the Radiologist. The Eliases had spent four years in England working mainly in Leicester and Cardiff and had happy memories of this time. They entertained me in their home and also took me on a very pleasant excursion to a lake outside the town were fish are abundant. Amongst trees at the edge of the lake are rustic tables and a hut where the proprietor will cook you freshly caught fish. These large fish looked like a big meal when set in front of you but their soft tender flesh dissolved in the mouth and with the salad, loaves and beer that Dr. Elias had brought with her made a memorable meal.

The Aswan Dam seems to have been a mixed blessing to Egypt, and expectations have not been fulfilled. The lake above the Dam, Lake Nasser is gradually silting up; the land which used to be inundated annually with a silt-bearing flood and was amongst the most fertile in the world, now requires fertilisers. The turbo-electric generators are failing to provide the country with all the cheap electricity that was hoped for and, last but not least, Bilharzia, the scourge of Egypt is on the increase. It is not so much in the Nile itself that the water snails which are hosts to the Shistosoma abound, as in the irrigation canals spreading out from it into the countryside. These canals are now filled to a constant level with stagnant water and the snails and the parasites flourish as never before. Each time I visited Professor Guirguis outpatient clinic three or more patients with bladder symptoms would be kept to the end for cystoscopy. This was done on the ordinary examination couch. The patient moved right up to the end of the couch with his heels and buttocks over the edge. The urethra was anaesthetised with local anaesthetic and the cystoscope slipped in. Irrigation was provided from a douche can held by a boy—simplicity itself. An immediate diagnosis was usually forthcoming from the very characteristic appearance of the pseudo-tubercles or 'double pearls' in the base of the bladder. Fortunately effective anti-bilharzial drugs are available, but the bladder tumours that result from long standing disease are as intractable as ever.

For my last weekend Dr. Attillah took me to Hurghada on the Red Sea, a small resort developing around an Oceanological Institute and a luxury hotel. The museum there contains fascinating examples of the strange creatures that inhabit this tropical sea including many varieties of shark such as the hammer head, the sword fish, the huge ray manta, and perhaps strangest of all the Dugong with a nearly human face, the mermaid of legend.

Space does not permit further recollection of much else of interest. My main impression of this extraordinary country a thousand miles long and a few miles wide where it never rains and all life comes from the river Nile is of dusty heat, marvellous monuments of incredible antiquity, and ancient methods of agriculture still effective, perhaps more so than the modern ones which are being introduced. In the hospital scene at Sohag there is a small community of dedicated, very hardworking doctors, trained to European standards and achieving work of a high standard despite a lack of the technical infrastructure, nursing skill and administrative competence that we take for granted. It was particularly gratifying that so many of them held higher degrees from our British Royal Colleges and recollected with pleasure the years they had spent with us and kindnesses they had been shown. I was very touched indeed by the kindness they showed me in return.

